# Characterization of sequences in human TWIST required for nuclear localization

**DOI:** 10.1186/1471-2121-10-47

**Published:** 2009-06-17

**Authors:** Shalini Singh, Anthony O Gramolini

**Affiliations:** 1 Department of Physiology, Charles H. Best Institute, University of Toronto, 112 College Street, Toronto, Ontario, M5G 1L6, Canada

## Abstract

**Background:**

Twist is a transcription factor that plays an important role in proliferation and tumorigenesis. Twist is a nuclear protein that regulates a variety of cellular functions controlled by protein-protein interactions and gene transcription events. The focus of this study was to characterize putative nuclear localization signals (NLSs) ^37^RKRR^40 ^and ^73^KRGKK^77 ^in the human TWIST (H-TWIST) protein.

**Results:**

Using site-specific mutagenesis and immunofluorescences, we observed that altered TWIST^NLS1 ^K38R, TWIST^NLS2 ^K73R and K77R constructs inhibit nuclear accumulation of H-TWIST in mammalian cells, while TWIST^NLS2 ^K76R expression was un-affected and retained to the nucleus. Subsequently, co-transfection of TWIST mutants K38R, K73R and K77R with E12 formed heterodimers and restored nuclear localization despite the NLSs mutations. Using a yeast-two-hybrid assay, we identified a novel TWIST-interacting candidate TCF-4, a basic helix-loop-helix transcription factor. The interaction of TWIST with TCF-4 confirmed using NLS rescue assays, where nuclear expression of mutant TWIST^NLS1 ^with co-transfixed TCF-4 was observed. The interaction of TWIST with TCF-4 was also seen using standard immunoprecipitation assays.

**Conclusion:**

Our study demonstrates the presence of two putative NLS motifs in H-TWIST and suggests that these NLS sequences are functional. Furthermore, we identified and confirmed the interaction of TWIST with a novel protein candidate TCF-4.

## Background

TWIST1 is a basic helix-loop-helix (bHLH) transcription factor [1] which forms either homo-or-heterodimers with other bHLH proteins to bind to a core E-box (CANNTG) sequence on the promoter region of target genes through the basic region [2]. Twist is necessary for the development of the mesoderm [1], cell type determination and differentiation during myogenesis [3], neurogenesis [4], cardiogenesis [5] and also required for formation of the head mesenchyme, somites and limb buds [6]. Twist loses its function as a negative modulator during the differentiation of separate mesodermal layers, myogenesis, osteogenesis or neurogenesis [7,8]. Twist has also been implicated in neural tube closure and null mice mutants are embryonic lethal at E10.5, whereas TWIST is essential for normal development and promotes autosomal dominant defects characterized by minor skull and limb anomalies in humans [6]. Mutations in TWIST1 result in Saethre-Chotzen Syndrome (MIM #101400, SCS), known as an autosomal dominant craniosynostosis [9,10]. In addition, mutations in the helix domain of the *TWIST1 *gene can cause subcellular mislocalization and increased degradation of its protein product [11]. Twist also acts as a key regulator of metastasis, and overexpression of TWIST1 in subsets of sporadic human breast cancer promotes epithelial to mesenchymal transition through down-regulation of E-cadherin which was confirmed in a murine breast tumor model [12].

Potential functions of TWIST1 are not well defined. Previous studies have shown that Twist functions as a transcriptional repressor and is regulated by its dimerization with other bHLH-containing transcriptional factors. For instance, post-translational modifications such as phosphorylation can alter the dimerization preferences of Twist, promoting either homodimer or heterodimer formation [13]. The Twist heterodimer can also act a transcription repressor, whereas Twist homodimer acts as a transcription activator in *Drosophila *mesoderm development and in human cranial suture patterning [14]. In order for a protein to function as an activator and/or repressor of transcription of a target gene, efficient nuclear localization is essential [15]. Directed nuclear entry requires the presence of nuclear localization signals (NLSs) that recognize and associate with the nuclear import receptors. Nuclear localization signals (NLSs) mediate active transport of protein into the nucleus selectively and efficiently [16]. Therefore, identification and functional characterization of TWIST NLSs would represent a necessary and important step in understanding the regulation of TWIST and its interaction with other bHLH proteins.

In this study, we identified two NLSs present at the N-terminal region of human TWIST rich in basic amino acids lysine and arginine. Using site directed mutagenesis and immunofluorescence assays, we studied the role of these two NLSs in nuclear localization. Yeast-two-hybrid and immunoprecipitation assays were also performed to identify TWIST-interacting proteins and gain a greater understanding of the mechanism responsible for H-TWIST localization and function.

## Results

### Comparison of various Twist proteins

**Figure 1:**
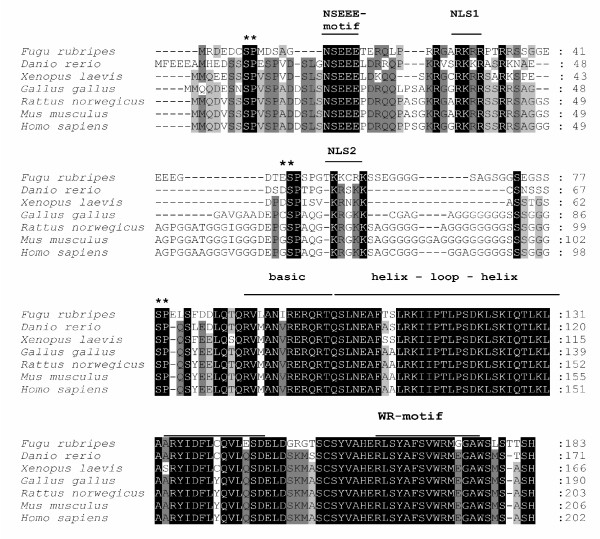
**Protein sequences comparison among vertebrate TWIST family members**. Alignment of amino acid sequences encoded by the Twist gene from different species showed beside the well known basic Helix-Loop-Helix (bHLH) motif, conservation in four additional protein regions. NLS1 and NLS2 mark putative nuclear localization signals. Gaps are indicated as hyphens. Black boxes indicate identical amino acids which are present in all species. Grey boxes are present in identical amino acid in six species and light grey boxes in five species, respectively. Alignment was generated by ClustalW.

H-TWIST contains 202 amino acids [17]. A sequence comparison of human, mouse, rat, chicken, claw frog, zebra fish and fugu twist proteins based on homology [18-21], identified five highly conserved domains in twist proteins. Besides a novel NSEEE-domain (aa 19–23), two potential NLSs, NLS1 and NLS2 found at the N-terminal region of the TWIST protein present at positions 37–40 (RKRR) and 73–77 (KRGKK), respectively with unknown function. These proteins share 80–100% homology from different vertebrates, more specifically 93% identity in the bHLH regions, 100% in NLS1, 80% in NLS2, and 100% in NSEEE and WR domains. It is already well known that in H-TWIST, HLH region present on amino acid position 121–160 is necessary for protein dimerization and the basic amino acid region present at the position 108–120 is required for DNA binding (Fig. 1).

### Subcellular-localization of H-TWIST-NLS mutants

Localization of many transcription factors has been shown to be an important mechanism in the regulation of protein functions. Transcription factor proteins are nuclear proteins able to interact with gene regulatory sequences to activate gene expression. To facilitate monitoring of the cellular localization of TWIST, we generated a c-myc-TWIST fusion expression construct and mutated the putative NLS motifs shown in Table 1, using site directed mutagenesis. For these experiments, we transfected cells with expression plasmids harbouring the gene for wild-type TWIST or TWIST mutated NLS motifs.

**Table 1:** Subcellular localization of TWIST^wt ^and NLS mutants.

**Clone Name**	**NLS1**	**NLS2**	**Nucleus**	**Cytoplasm**
TWIST^wt^	RKRR	KRGKK	**+**	
K38R	R**R**RR	KRGKK		**+**
K73R	RKRR	**R**RGKK		**+**
K76R	RKRR	KRG**R**K	**+**	
K77R	RKRR	KRGK**R**		**+**

The mutated amino acids are in bold and underlined. (+), indicates presence of protein within that subcellular compartment determined by immunofluorescence assays.

**Figure 2:**
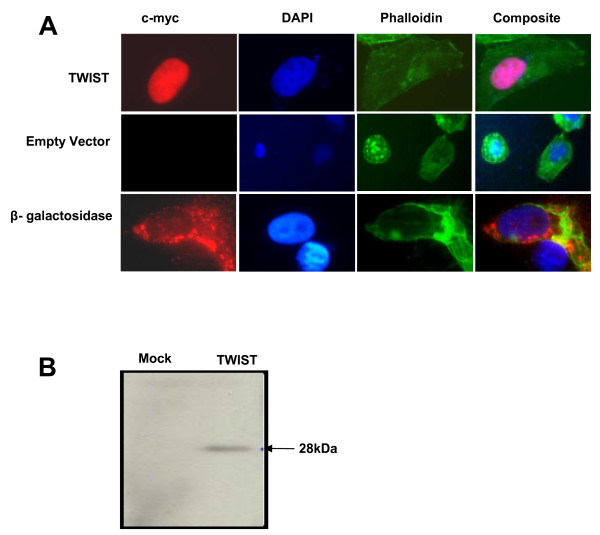
**Subcellular-localization of TWIST full length fusion protein was monitored in transfected U2-OS cells**. Transfection with empty vector (pCMV) was used as a negative control and a c-myc β-galactosidase was the positive control. Expression of c-myc-TWIST proteins was detected by fluorescence using anti-c-myc antibody. DAPI staining (blue) indicates the nucleus, while phallodin (green) represents the actin cytoskeletal staining. (b) Expression level of TWIST wild type protein shown by Western blotting with anti-c-myc antibody.

The recombinant TWIST^WT ^protein localized exclusively to the nucleus of transient transfected cells, in agreement with a previous report on H-TWIST localization [11] (Fig. 2A). Transfection with pCMV alone was used as a negative control. No c-myc signals were detected with empty vector (Fig. 2A). As a positive control, we fused c-myc with β-galactosidase (β-Gal). The β-Gal-cmyc fusion protein was seen predominantly in the cytoplasm [22] (Fig. 2A).

Expressions of the TWIST fusion proteins were also examined by cellular fraction and immunoblotting. A single 28-kDa protein band was recognized for c-myc-TWIST^WT ^by an anti-c-myc antibody (Fig. 2B). pCMV empty vector transfected in cells was used as a negative control along with c-myc-TWIST^WT ^fusion construct in our first set of experiments. No signal was detected in cells transfected with the negative control plasmid (Fig. 2B).

**Figure 3:**
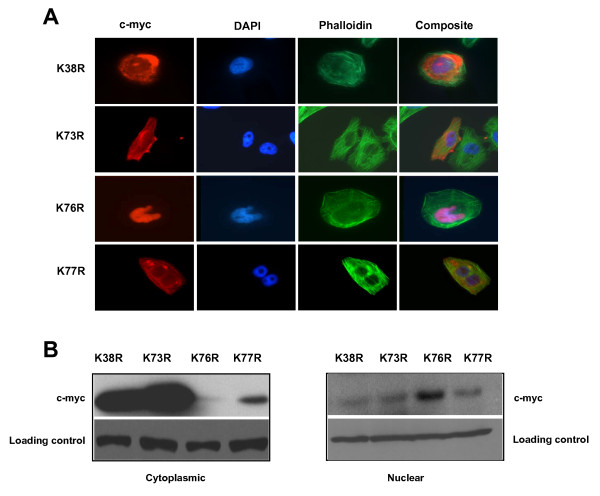
**Mutational analysis of putative NLSs in H-TWIST were observed in U2-OS cells, the expression of c-myc-TWIST mutants were detected by fluorescence using anti c-myc antibody**. Nuclei were stained with DAPI (blue), and phalloidin (green) representing the actin cytoskeletal. Site-directed mutagenesis generated a single amino acid change, K38R, which was sufficient to block TWIST nuclear localization and, therefore, redistribute approximately 92% the mutant protein to the cytoplasm. Mutations in TWIST^NLS2 ^at positions K73R and K77R also resulted in cytoplasmic expression, as approximately 83% and 87% of transfected cells had very low levels in the nucleus. (b) Analysis of expression levels of TWIST mutant constructs in cytoplasmic and nuclear fractions of transfected cells. Equivalent loading was determined by GAPDH and α-actin expression levels.

Classical NLS motifs are composed of a basic amino acid stretch (K/R)4-6 preceded by a Gly, Pro, or an acidic amino acid residue [27]. The N terminal of M-TWIST which is an analogue of H-TWIST binds to p300/pCAF and decreases its histone acetyl-transferase activity [24]. Alignment of amino acid sequences encoded by the Twist gene from different species predicts two putative NLSs within H-TWIST at the N terminal with the most conserved segment containing lysine residues. Therefore, our hypothesis was that the lysine residues in the NLS motif might be essential in nuclear localization. Mutation in the NLS1 site strongly abrogated the nuclear localization of the fusion protein as the TWIST^NLS1 ^mutant (K38R) was found to be no longer enriched in the nucleus but showed high expression in the cytoplasm of 93 out of 100 transfected cells (Fig. 3A). This provides experimental evidence that TWIST-NLS (37–40 aa) is functionally significant for nuclear localization. Mutation in TWIST^NLS2 ^at position K73R, and K77R also resulted in cytoplasmic expression (88% and 83%, respectively) as these transfected cells showed very low levels in the nucleus. In contrast, the subcellular localization of TWIST^NLS2 ^K76R mutant was only observed in the nucleus, at levels consistent with the TWIST^WT ^(Fig. 3A, 3B).

In biochemical experiments, TWIST NLS mutant proteins were in cytoplasmic fractions of transfected cells by immunoblot analyses. In these experiments, there were high levels of expression for the K38R and K73R mutants in the cytoplasmic lysate (Fig. 3B), consistent with our microscopy (Fig. 3A). We detected very low levels of the K76R mutant in the cytoplasm; a finding entirely consistent where the majority of staining is seen restricted to the nucleus. Interestingly, K77R showed expression in the cytoplasm, although our imaging results identified that it was not restricted to the nucleus. These results would indicate that this mutant that loses its nuclear targeting is either not soluble or expressed perhaps in intracellular organelles not solubilized by our protocol.

### Rescue of mislocalization of TWIST mutants by co-transfection with heterodimer partner E12

**Figure 4:**
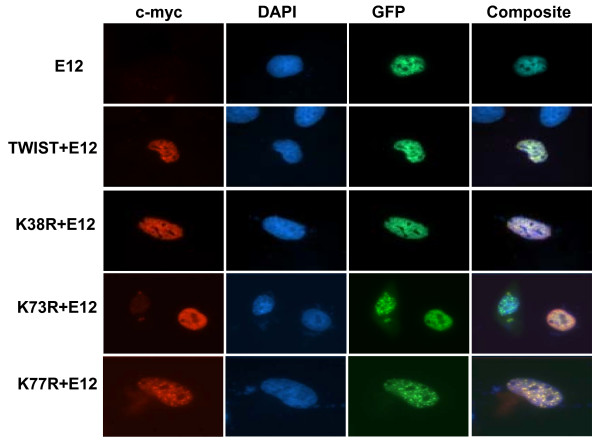
**Rescue of mislocalization of TWIST mutant**. C-myc expression TWIST and TWIST mutant constructs with E12-eGFP were visualized with digital imaging. E12-eGFP construct expression was observed in the nucleus. E12-eGFP constructs were co-transfected with K38R, K73R and K77R mutants indicate the co-localization of E12 with TWIST^NLS ^mutants into the nucleus.

bHLH transcriptional factor proteins are able to form either a homodimer or heterodimer with other bHLH protein for DNA binding. Given this property, we selected E12, a bHLH protein which forms a heterodimer with other bHLH proteins and also has been shown to interact with Twist [11]. Co-localization of TWIST with E12 protein was studied to determine the recovery of localization of TWIST NLS mutants in the nucleus. For this, E12 cDNA was subcloned into the eGFP-N1 vector in the same GFP reading frame and protein expression was examined in cells using fluorescence microscopy. In a control experiment, eGFP vector alone was transiently transfected into the cells and exhibited non-specific distribution of fluorescence protein throughout the cell (data not shown), while in contrast eGFP-E12 interaction was predominantly localized in the nucleus (Fig. 4).

In order to investigate direct interactions between TWIST and E12, the TWIST^WT ^and TWIST^NLS1 ^mutant with eGFP-E12 were transiently transfected into the cells. Here, we observed that the coexpression of TWIST^NLS1 ^mutant K38R with E12 compensates for the mislocalization of this construct and results in the restoration of nuclear expression of the TWIST mutant. The same co-expression results were obtained using K73R and K77R with E12 (Fig. 4). Nuclear localization could be observed despite the non-functional NLSs.

### Screening for TWIST interacting partners using yeast two hybrid assay

To gain additional insights into the roles of TWIST domains, we were interested in the identification of proteins interacting with TWIST, and thus utilized a yeast two-hybrid screening assay (Materials and Methods). We identified a TWIST-associated protein called Transcription factor 4 (TCF-4) also known as immunoglobulin transcription factor-2 (ITF-2), SL3-3 and enhancer factor-2 (SEF-2), (MIM 602272; Genbank accession NM_003199) (Table 2).

**Table 2:** TWIST interacting proteins were identified by yeast two hybrid experiments.

**Clone**	**Result**	**Remark**	**Gene Bank Acc.No**.
TW1-2	mRNA Splicing factor 2	Nuclear protein	L04636
TW1-8	TCF4/SEF2-1A Transcription factor	HLH protein	M74718
TW1-10	pre mRNA splicing factor 2	Nuclear protein	L04636
TW1-11	pre mRNA splicing factor 2	Nuclear protein	L04636
TW1-15	KIAA1376-protein	unknown	XM_033042
TW1-16	KIAA1376-protein	unknown	XM_033042
TW1-18	Clusterin	Sulfated-glycoprotein	BC010514

Yeast two hybrid positive clones were identified by blast sequence analysis. Our clone ID, putative interacted protein name, subcellular localization and accession numbers are shown.

**Figure 5:**
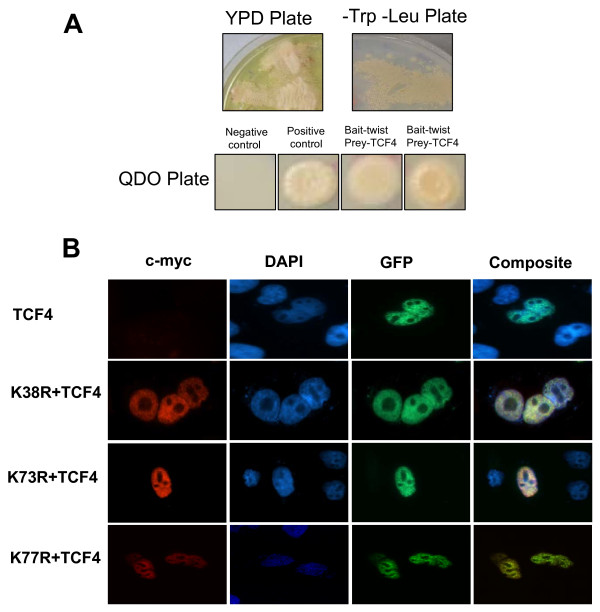
**Yeast mating to identify TWIST protein interactions**. (A) Yeast cells transformed with the indicated combinations of bait (pGBKT7-TWIST) and prey (pGAD-TCF-4) plasmids on YPD plate. Co-transformed yeasts were spotted on -Leu-Trp plate representing the successful cell mating (irrespective if fusion proteins interact); by colony lift assay. TWIST and TCF4 protein interaction was visualized on selective QDO Plates which contains -Leu-Trp-Met-His (quadrate drop out) reporter plates containing 1 mM 3-aminotriazole (3-AT). In this assay, p53/pGBKT7 clone transformed into AH109 mated with Y187 strain containing T7-antigene in pGADT7-vectors was used as a positive control and p53/pGBKT7 in AH109 mated with LamC/pGADT7 which was transformed into Y187 used as a negative control to confirm specificity of the interaction. (B) NLS rescue assay to study the ability of TCF-4 to assist in the nuclear localization of TWIST^NLS1 ^mutants. Co-transfection of K38R, K73R, and K77R with TCF4 results in the restoration of TWIST mutants in the nucleus.

The interaction between TCF-4 and TWIST was confirmed in yeast by co-transformation TCF-4 plasmid and the TWIST bait plasmid on nutritional quadrate dropout plates with 1 mM 3-amino triazole to determine reporter gene activation. In this assay, p53/pGBKT7 clone transformed into AH109 mated with Y187 strain containing T7-antigene in pGADT7-vectors was used as a positive control, and p53/pGBKT7 in AH109 mated with LamC/pGADT7 transformed into Y187 was used as a negative control to confirm the specificity of the interaction. Only the yeast cells containing both TCF-4 and TWIST were able to grow on the nutritional dropout plates, suggesting the interaction between TCF-4 and TWIST was the result of specific binding (Fig. 5A). Similar results for β-galactosidase activity were also observed in all yeast positive clones (data not shown).

### Confirmation of the TCF-4 and TWIST interaction by NLS rescue assay

As discussed above, TWIST is a nuclear protein that forms dimers with other HLH or bHLH protein, and mutations in TWIST NLSs result in nuclear mislocalization. Since we hypothesized that the co-transfection of TCF-4 with NLS deficient TWIST will restore protein localization to the nucleus, we performed additional NLS rescue assays, using c-myc TWIST expression constructs and co transfected with a TCF4-GFP construct. The empty GFP was distributed throughout the cell (data not shown), whereas TCF4-GFP expression was predominantly localized to the nucleus of cells (Fig. 5B). In this assay, TWIST^NLS1 ^mutant which was unable to be transported in the nucleus was cotransfected with TCF-4 to check whether the lack of a functional NLS could be compensated by heterodimerization and subsequent co-import of the two proteins. Co-localization of both proteins in the nucleus was detected in the cells co-transfected with both TWIST^NLS1 ^mutant and TCF-4 constructs (Fig. 5B). The same co-expression results were observed using K73R and K77R with TCF-4 (Fig. 5B).

**Figure 6:**
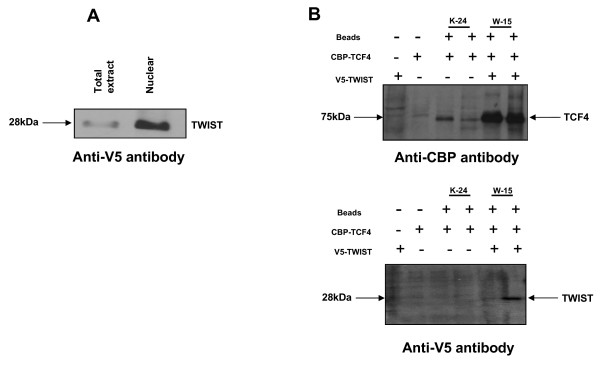
**Validation of the interaction of TWIST with TCF-4 using immunoprecipitation assays**. (A) Expression of wild type TWIST was shown by Western blotting using the anti-V5 antibody, (B) HEK293 cells were co-transfected with plasmids expressing V5-HIS-TWIST and CBP-TCF-4. Two different CBP antibodies were used (clone ID; K-24 and W-15). W-15 was used for the immunoblot. IgG beads with protein and antibody, and beads without antibody were used as positive and negative controls, respectively. Nuclear extracts were immunoprecipitated with anti-CBP tag antibodies (K-24 and W-15) bound resin, and the precipitated proteins were detected using anti-V5 antibody.

Furthermore, having identified TCF-4 as an interacting protein for TWIST in the yeast two-hybrid assay, we examined the ability of TWIST to interact with TCF-4 protein in mammalian cells by performing immunoprecipitation from nuclear extract of HEK293T cells transfected with full length V5- tagged TWIST in the presence of CBP- tagged TCF-4. Immunocomplexes precipitated with anti-CBP antibody were resolved by SDS-PAGE and Western blotting using anti-V5 antibodies identified bands corresponding to the expected sizes of the TWIST showed the interaction between these two proteins (Fig. 6B). For these pull down experiments, we used two different CBP antibodies (clone IDs; K-24 and W-15) to differing success rates. We determined that although Ab K-24 worked to some extent, the best affinity pulldowns were observed with the W-15 antibody, with at least a 5-fold greater purification observed (Fig 6B). IgG beads with protein and antibody, and beads without antibody were used as positive and negative controls, respectively. Extracts immunoprecipitated (IP) with theses anti-CBP tag antibodies were then probed using anti-V5 antibody to determine whether V5-conjugated TWIST was co-precipitated under these conditions. No TWIST was seen in the control conditions, but a clear specific band representing TWIST was seen in the cotransfected cells immunoprecipitated with the W-15 antibody.

## Discussion

In this study, we identified two putative NLSs at the N-terminal region of TWIST and found that these NLSs mediate the expression of TWIST in the nucleus. An evolutionary alignment of Twist proteins amino acid sequences encoded from different species showed five different highly conserved regions NLS1, NLS2, NSEEE at N-terminal, WR at C-terminal and bHLH DNA binding domain. The HLH region is necessary and sufficient for protein dimerization [23]. Besides having two putative nuclear localization signals, the N-terminal of M-Twist which is an analogue of H-TWIST can also bind to p300/pCAF and decrease its histone acetyl-transferase activity [24]. Moreover, the C-terminus of Twist possesses a WR domain which is a RunX2-binding "Twist Box" and inhibit the RunX2 function during skeletal development [25].

### Nuclear localization signals in TWIST

The regulation of protein transport into the nucleus of a eukaryotic cell is mediated by specific nuclear localization signals (NLSs) recognized by protein import receptors [26]. The NLS sequence consists of a cluster of basic residues, either monopartite or bipartite [27,28]. This sequence is subsequently recognized by the heterodimeric import receptor complex comprising importin α and importin β [16]. Importin α is an adapter protein that consists of a small N-terminal β-binding domain. Importin β does not directly interact with the NLS cargo but acts to direct importin α to the nuclear pore [29]. The nucleocytoplasmic trafficking of large molecules is normally mediated by classical NLSs [30] and necessary for normal physiology and cellular trafficking. It might be that additional NLS sites exist in other domains of the proteins. Here, we show that N-terminal of H-TWIST contain 2 NLS motifs which are required for nuclear transport of this protein.

We mutated both of the predicted NLS lysine residues to determine their potential role in the importation of TWIST protein. The NLS1 mutant constructs were observed in the cytoplasm indicating that TWIST^NLS1 ^is a functional NLS, which governs the nuclear import of TWIST. Previously, similar results were made for murine Dermo-1, a bHLH protein encoded by the *TWIST2 *gene, in which engineered replacement of the equivalent NLS (also containing the RKRR sequence) by four alanine residues impaired nuclear import [31]. It is of particular interest that a novel mutation in the NLS1 of TWIST protein reported in SCS patients at C115G, resulting in an Arg39Gly mutation was not restricted to the nucleus, but found expressed in both nucleus and cytoplasm in COS-7 cells [21]. These results are entirely consistent with our findings here, identifying this region as a core NLS motif.

We also examined the functional role of NLS2 by mutagenesis and found that NLS2 likewise NLS1, also played an essential role in the nuclear import of TWIST protein. Although in this study we focused on the lysine residues within the NLS1 and 2 motifs, it is also possible that other residues within these domains may also contribute to nuclear localization. From our findings, it is concluded that sequences at the 38, 73, and 77 amino acid positions within the NLS domains are critical residues. In contrast, the 76 amino acid does not appear to be critical for nuclear import.

### Heterodimerization of TCF-4 with TWIST and co-localization in the mammalian cells

H-TWIST belongs to a class of bHLH protein known to form stable heterodimers with members of class a bHLH transcription factors [2,32]. Interestingly, Twist and MyoD interactions may prevent the specific activation of target genes for osteogenic differentiation, which has been proposed to result in the molecular pathogenesis of the Saethre-Chotzen syndrome [8]. E12 also known as TCF-3, a bHLH protein, has been reported to interact with H-TWIST and this heterodimerization could compensate the nucleus localization of TWIST having mutations in bHLH motif in a co-transfected Cos7 cells [11]. We utilized a similar approach in the case of mutated TWIST-NLSs to confirm the expected nuclear location of the wild-type TWIST protein and the TWIST-NLS mutants with E12 and reported that E12 interaction with TWIST compensates the non-functional NLSs.

In yeast-two-hybrid screening, we identified several novel proteins interacting with TWIST including a gene encoding the class I bHLH protein TCF-4 (transcription factor 4, also known as ITF-2; and SEF-2). The *TCF-4 *gene is conserved in many species, its expression found in adult heart, placenta, skeletal muscle, lung where TWIST is also highly expressed [33-36]. TCF4 is a bHLH transcription factor and contains two alternatively spliced forms of mouse TCF4 (ITF-2), termed ITF-2A and ITF-2B, that differentially regulate MyoD activation [37]. An alignment of the N-terminal 83 amino acids with the N-termini of full-length E12 and full-length E47 and ITF-2B revealed that this domain is 51% identical among the three E type proteins and HLH domain was also highly conserved in all three proteins [38]. The high expression levels of TCF4 in the brain suggests that it may regulate the activity of some neuron and neuro-endocrinology specific promoters, including the tyrosine hydroxylase enhancer and the somatostatin receptor II promoter [39].

In this study, we confirmed the heterodimerization of TCF-4 with TWIST in NLS rescue assays and *in vitro *binding assay. Since the NLS mutant TWIST is unable to localize to the nucleus, this study illustrates that TWIST can form a functional complex together with overexpressed TCF-4, similar to that observed with E12, and that these interactions can restore nuclear localization despite the mutation in NLSs.

## Conclusion

It has already been reported that protein transcription factors may possess more than one NLS [40]; presumably a multitude of NLS may increase the rate of protein factor-transporter protein interactions [41]. Our study demonstrates the presence of two putative NLS motifs in H-TWIST and suggests that these NLS sequences are functional. These two NLSs may also operate in a cooperative manner for more efficient nuclear translocation. We also conclude that the mislocalization of NLS mutated construct could be restored with other bHLH proteins through heterodimerization with H-TWIST.

## Methods

### Plasmids construction

TWIST open reading frame (ORF) was amplified by PCR from human genomic DNA using oligonucleotides CGCGGATCCGCGATGATGCAGGACGTGTCC and CCGGAATTCCGGCTAGTGGGACGCGGACAT, containing *BamHI *and *EcoRI *sites respectively. After *BamHI *and *EcoRI *digestion, it was ligated into a pCMV mammalian expression vector, which contains a *c-myc *epitope. The E12-ORF was amplified by the PCR using oligonucleotides CCGGAATTCCGGATGGCGCCTGTGGGCACA and CGCGGATCCGCATGTGCCCGGCGGGGTTGT respectively. This was subcloned in Topo TA cloning vector. After *EcoRI *restriction from Topo vector the E12 ORF subcloned in eGFP-N1 vector in the same GFP reading frame. β-galactosidase open reading frame was digested with *BamHI *and *XhoI *from pcDNA4/myc-His/lacZvector and subcloned in pCMV-cmyc mammalian expression vector. For yeast two hybrid assay the TWIST-ORF was amplified from the human genomic DNA using primers CCGGAATTCCGGCTATGATGCAGGACGTGTCC and CGCGGATCCGCGCGTGGGACGCGGACATG 3' containing *EcoRI *and *BamHI *sites respectively. After *EcoRI *and *BamHI *digestion, it was ligated into a pGKT7 yeast bait vector, which contains a GAL4 DNA binding domain. For the NLS rescue assay, TCF4-ORF was amplified using oligonucleotides CCGGAATTCCGGATGCATCACCAA and CGCGGATCCGCGCATCTGTCCCAT, containing *EcoRI *and *BamHI *restriction sites, respectively. After digestion with *EcoRI *and *BamHI*, TCF4-ORF was ligated into mammalian expression eGFP-N1 vector. For immunoprecipitation experiments, TWIST-cDNA was subcloned into V5-HIS tag vector (Invitrogen) using clonase, and TCF4 ORF clone was subcloned into the pcTAP mammalian expression vector (Stratagene). All constructs were fully sequenced.

### Site-directed mutagenesis

*In vitro *mutagenesis of double stranded DNA templates was performed as described [42]. PCR was performed using 50 ng of TWIST plasmid DNA and 125 ng of each primer with Pfu Turbo polymerase using Site-Directed Mutagenesis Kit (Stratagene, USA) according to the manufacturer's instructions. The final plasmids were sequenced to confirm the presence of the mutation and the absence of PCR artifacts. Plasmids expressing c-myc-tagged TWIST with an intact and mutated NLS sequences are shown in Table 3.

**Table 3:** Oligonucleotides used for mutagenesis reactions to alter NLSs sequences in TWIST.

**Amino acid substituted in NLSs**	Primer Sequence
K38R	5'CGCGGGGGACGCAGGCGGCGCAGCAGC3' 5'GCTGCTGCGCCGCCTGCGTCCCCCGCG3'
K73R	5'CCCAGGGCAGGCGCGGCAAGAAGT3' 5'ACTTCTTGCCGCGCCTGCCCTGGG3'
K76R	5'CAAGCGCGGCAGGAAGTCTGCGG3' 5'CCGCAGACTTCCTGCCGCGCTTG3'
K77R	5'GCGGCAAGAGGTCTGCGGGCT3' 5'AGCCCGCAGACCTCTTGCCGC3'

K, lysine; R, arginine

### Cell culture and transfection assay

Cell chamber slides were seeded with 1.5 × 10^5 ^U2-OS (Human osteosarcoma, ATCC) cells, and grown over night in DMEM (Gibco BRL) supplemented with 10% FBS containing 100 μg/ml penicillin and streptomycin. To select a cell line several factors were considered including growth conditions, proliferation ability and ease of transfection. Considering all factors together, several cell lines were used here including OHS, CCL-136, COS7 and U2-OS. Finally, we selected U2-OS cells for further studies. The cells were transiently transfected with 500 ng of plasmid by using Effectene (QIAGEN) according to the manufacturer's instructions.

### Immunofluorescence assays

Culture chamber slides were fixed with 4% paraformaldehyde for 20 min at 25°C, and permeabilized with 0.2% Triton X-100 for 5 min. Subsequently, these slides were incubated for 1 h at 37°C with the mouse monoclonal anti-c-myc primary antibody (Molecular Probes, 1:200 dilution). Slides were further incubated in secondary antibody (Texas Red conjugated goat anti-mouse IgG; Molecular probes, 1:1000 dilution) for 1 h at 37°C. The slides were washed and incubated with phalloidin for 20 min at 25°C and incubated with DAPI at 25°C for 3 min and then mounted by using Vector Shield (Vector Laboratories, CA, USA).

### Protein extraction and Western immunoblot analysis

To confirm the expression of TWIST and TWIST NLSs in transfected U2-OS cells for immunoblot analysis, cytoplasmic fractions were isolated from these transfected cells by scraping after 24 h of incubation and then centrifuged for 5 min. The cells were lysed with NP40-containing lysis buffer (10 mM Tris, pH 7.4, 10 mM NaCl, 5 mM MgCl_2_, 0.5% NP-40) to disrupt the cell membrane and then the cell lysate was centrifuged at 500 × g for 5 min at 4°C. The supernatant (cytoplasmic fraction) was removed and the pellet (nuclear fraction) was resuspended in NP-40 containing cell lysis buffer. Proteins were denatured by boiling in sample buffer, separated on 12% SDS-PAGE and then transferred onto the PVDF membrane (Immobilon TM-PSQ, Millipore) and blocked overnight in 5% non-fat powdered milk in TBST (10 mM Tris-HCl pH7.5, 100 mM NaCl, 0.1% (v/v) Tween 20). Mouse monoclonal anti c-myc antibody (1:1000 diluted in TBST) (Molecular probe) used for protein detection. Peroxidase conjugated goat anti-mouse IgG (1:10,000 diluted in TBST) (Sigma, Missouri, USA) was used as a secondary antibody.

### Yeast-two-hybrid assay

The full-length TWIST was used as a bait protein. After transformation of pGBKT7-TWIST into yeast strain AH109, expression of the fusion protein GAL4-BD-TWIST was confirmed by PCR (as described Clonetech Manual). Yeast strainY187 with pretransformed human placental cDNA library was mated with AH109 strain containing pGBKT7-TWIST, and the cells were then plated onto SD/-Ade/-His/-Trp/-Leu plates to screen the expression of ADE2 and His3 genes (Fig 5A). Yeast colonies expressing both of the two genes were subjected colony-lift filter assays to check the LacZ gene expression. Consequently, we isolated 24 positive colonies with β-galactosidase activity following the procedure previously described [43] and sequenced the plasmids.

### Pulldown assays with IgG agarose beads

Mammalian expressed, affinity tagged proteins were purified from transfected cells using IgG agarose beads. Fifty microlitre of a 50% (v/v) agarose beads slurry containing 10–50 μg of purified V5-His-tagged protein were incubated with 100 μg of purified CBP fused protein in a total volume of 200 μl TBS containing 0.1% (v/v) BSA. Following a 2 h incubation with continuous rotation at 4°C, the agarose beads were washed 5 × 10 min at 4°C with the same buffer and 2 × 5 min in TBS without BSA. Agarose beads were mixed with an equal volume of 2× loading buffer and bound proteins were eluted by incubation for 1 h at 4°C and were subsequently separated by SDS-PAGE gels.

## Authors' contributions

SS performed all of the experiments and drafted the manuscript. AOG defined the research theme, assisted in experimental design and edited the manuscript. All authors read and approved the final manuscript.
